# 

**Published:** 1913-03-08

**Authors:** 


### The Supply of Drugs for Insured Persons.

A COMMITTEE OF INQUIRY APPOINTED.

We are officially informed that a Committee has been appointed, consisting of Mr. James Smith Whitaker, M.R.C.S., L.R.C.P., Chairman of the National Health Insurance Commission (England), Mr. Walter Davies, Mr. James Crawford Ledlie, of the Privy Council Office, Mr. Claud Schuster, of the National Health Insurance Commission (England), and Dr. Lauriston Elgie Shaw, M.D., F.R.C.P., to inquire and report whether, having regard to the interests of insured persons in obtaining an efficient and rapid supply of drugs, medicine, and appliances, and to the conditions under which those articles were supplied before the passing of the National Insurance Act, 1911, any alteration is necessary in the conditions laid down by Section 15 (5) of the National Insurance Act in respect of the matter. All communications in regard to the Committee should be addressed to the Secretary, Mr. James Rae, at the National Health Insurance Commission (England), Buckingham Gate, London, S.W.

### THE LATE BARON ILKESTON.

The estate of the Right Hon. Balthazar Walter, First Baron Ilkeston, F.R.C.P., M.D., is valued at £18,784 net. Apart from family bequests, the most important provision is that by which £800 is left in trust to the authorities of Durham University to found a "Winifred Foster" Studentship for a woman student who requires help to maintain herself at the University. This bequest is in memory of Lord Ilkeston's late daughter, who was a student at Durham University.

Dr. Edward Henderson, late of Shanghai, has died in London, aged seventy-two. Dr. Henderson graduated M.D. at Edinburgh in 1864, and became a F.R.C.S. Edin. in 1879. He was surgeon-general of the hospital in Shanghai, medical officer of the British Consulate, municipal medical officer and health officer.

### CONTRACTING OUT IN MIDDLESEX.

Doctors were present for the first time last Monday at a meeting of the Middlesex Insurance Committee. It was proposed that the committee should permit hospital asylum staffs and insured persons desiring a particular system of treatment to make their own arrangements.

Dr. Lowry urged the committee to allow a person who had been treated by a non-panel doctor to continue to be treated by him. He argued that when the committee came to deal with the residue of insured persons who had not been able to get upon the lists they would experience some difficulty. He denied that the non-panel doctors were trying to wreck the Act.

Mr. Warnford Davis pointed out that of the 306,000 insured persons in the county 130,000 had not yet selected their doctors.—Mr. Kelland, the chairman of the general purposes committee, said that the recommendations made to the committee did not prevent the extension of contracting out.—Dr. Lowry said if he accepted the gasworkers of Brentford their presence in his surgery would drive the better class of patients away.—The recommendations were agreed to.

The committee approved of advance payments at the rate of 7d. for each insured person to the 511 doctors on the panel. The largest sum thus paid to one practitioner was £62 12s. 5d. It was pointed out that this sum represented 2,147 insured persons, and a member said, as will surprise no one who read Mr. E. B. Turner's remarks in The Hospital of February 8, that if there was an epidemic that doctor might find his hands too full.

### DOCTORS' WILLS.

Dr. William Howship Dickinson, M.D., F.R.C.P., of Tintagel, Cornwall, who died on January 9, aged eighty, left estate valued at £39,790 gross and £19,696 net. He bequeathed £1,000 to the Samaritan Fund of St. George's Hospital, to be known as the "Lee Dickinson Memorial Fund." Dr. Dickinson was a Fellow of the Royal College of Physicians and for some time examiner for the Royal Societies and for London, Cambridge, and Durham Universities.

### HONOUR FOR ONE OF THE FRENCH HOSPITAL'S STAFF.

Dr. Louis Vintras, physician to the French Hospital in London, and director of the French Convalescent Home at Brighton, has been made a Knight Grand Cross of the Order of the Knights Hospitallers of St. John in Spain.

### The County Hospital, Winchester.

The annual court of governors of the Royal Hampshire County Hospital, Winchester, was held on Wednesday last week, when, in the absence of the Duke of Wellington, Mr. J. C. Warner, chairman of the committee of management, was appointed chairman of the meeting. As the Duke of Wellington did not seek re-election, upon the motion of Sir William W. Portal, Bart., the Earl of Northbrook was appointed chairman for the ensuing year. As the ordinary income amounted to £6,895, while the ordinary expenditure reached the figure of £7,832, leaving an excess of expenditure of £937. It is interesting to note that legacies amounted to £1,165, in consequence of which the total expenditure for the year has only exceeded the total income by the sum of £22. The in-patients for the year numbered 1,154, the out-patients 1,201. The average cost per bed occupied had been reduced from £69 7s. 2d. to £67 16s. 11d. The court received the annual report with an expression of satisfaction. The Chairman reported that the patients' lift, the cost of which has been generously defrayed by Miss Burrell, of Botley, is now being installed, and voiced the thanks of the court to the donor. Her gift, it is felt, will undoubtedly add greatly to the comfort of the patients.

### GIFT TO CARDIFF HOSPITAL.

In quick response to Colonel Bruce Vaughan's recent appeal, Mr. George Birt, of Cardiff, has forwarded fifty guineas in the hope that others will follow in providing a sufficient sum of money to justify the board of management in opening the fifty remaining beds in the new wing. As Mr. Birt has been a member of the board for some time, he has had intimate opportunities of judging of the needs of the hospital.

### Appointments.

Mr. R. G. McEntire has been appointed fifth assistant medical officer at Cane Hill Mental Hospital.

Dr. A. F. Cameron, senior assistant medical officer assistant medical officer at Colney Hatch Mental Hospital.

Mr. R. B. N. Reade has been appointed as sixth assistant medical officer at Hanwell Mental Hospital.

Dr. A. F. Cameron, senior assistant medical officer at the South-Eastern Hospital, has been appointed medical superintendent at the Downs Sanatorium at a salary of £450 a year. Dr. J. E. Squire, tuberculosis officer under the London Insurance Committee, has been appointed as visiting specialist to direct and be responsible for the general treatment of the patients at a remuneration of £315 a year.

At a recent meeting of the board of management of the Manchester Royal Infirmary the following appointments were confirmed:—Messrs. G. C. Dixon, M.B., Ch.B. (Vict.), and L. W. Sparrow, M.B., Ch.B. (Vict.), house physicians; Messrs. C. F. White, M.B., Ch.B. (Vict.), and P. H. Midgley, M.B., Ch.B. (Vict.), senior house surgeons; Messrs. G. B. Buckley, M.R.C.S., L.R.C.P., and T. W. Martin, M.B., Ch.B. (Vict.), junior house surgeons.

Dr. S. N. Galbraith, M.B., Ch.B., D.P.H., has been appointed assistant tuberculosis officer under the Lambeth Borough Council. Dr. Galbraith is acting resident medical officer at Brompton Hospital, and was previously house surgeon at the General Hospital, Cheltenham, house physician at Brompton Hospital, and visiting physician at the Doncaster Corporation Sanatorium. The commencing salary of his office under the Lambeth Borough Council is £300 a year. There were fifteen applications for the appointment.

### Obituary.

The death is announced of Mr. Robert Falconer Macdonald, F.R.I.B.A. Though Mr. Macdonald was chiefly engaged on secular and collegiate buildings, he carried out in 1907 the Infants Hospital in Vincent Square, Westminster.

The death is also announced of Mr. James Francis Doyle, architect, who was associated with the late Mr. R. Norman Shaw, R.A., in important works. In 1900 he carried on the Central Home in Liverpool for the Victoria Diamond Jubilee Nurses' Institution, and in 1910 the new out-patients' department of Liverpool Royal Infirmary, at a cost of about £30,000.

### ORDER OF ST. JOHN OF JERUSALEM.

Among the new knights promoted in or appointed to the Order of the Hospital of St. John of Jerusalem in England we note His Highness Prince Alexander of Battenberg, Lieutenant-Colonel Thomas Horrocks Openshaw, and Colonel Clement Godson, M.D.

### PRESENTATION TO A LADY DOCTOR.

At the Council Offices, Acton, on the 25th ult., Mr. F. A. Everitt, secretary of the Acton Education Committee, on behalf of members of the Acton Municipal Officers' Association and the teachers in the service of the Acton Education Committee, presented to Dr. Lilian E. Wilson, late school medical inspector of school children at Acton, half a dozen silver-backed dressing-table requisites as a mark of esteem, on her leaving Acton to take up an important appointment on the medical staff of the Board of Education.

